# Effects of tributyltin on placental and reproductive abnormalities in offspring

**DOI:** 10.20945/2359-4292-2024-0186

**Published:** 2024-11-06

**Authors:** Charles S. da Costa, Hanin Alahmadi, Genoa R. Warner, Maria Tereza Nunes, Glaecir Roseni Mundstock Dias, Leandro Miranda-Alves, Jones B. Graceli

**Affiliations:** 1 Universidade Federal do Espírito Santo Departamento de Morfologia Vitória ES Brasil Departamento de Morfologia, Universidade Federal do Espírito Santo, Vitória, ES, Brasil; 2 New Jersey Institute of Technology Department of Chemistry and Environmental Science Newark NJ USA Department of Chemistry and Environmental Science, New Jersey Institute of Technology, Newark, NJ, USA; 3 Universidade de São Paulo Instituto de Ciências Biomédicas Departamento de Fisiologia e Biofísica São Paulo SP Brasil Departamento de Fisiologia e Biofísica, Instituto de Ciências Biomédicas, Universidade de São Paulo, São Paulo, SP, Brasil; 4 Universidade Federal do Rio de Janeiro Programa de Pós-graduação em Endocrinologia Faculdade de Medicina Rio de Janeiro RJ Brasil Programa de Pós-graduação em Endocrinologia, Faculdade de Medicina, Universidade Federal do Rio de Janeiro, Rio de Janeiro, RJ, Brasil; 5 Universidade Federal do Rio de Janeiro Instituto de Biofísica Carlos Chagas Filho Laboratório de Fisiologia Endócrina Doris Rosenthal Rio de Janeiro RJ Brasil Laboratório de Fisiologia Endócrina Doris Rosenthal, Instituto de Biofísica Carlos Chagas Filho, Universidade Federal do Rio de Janeiro, Rio de Janeiro, RJ, Brasil.; 6 Southern Illinois University School of Agricultural Sciences Animal Science Carbondale IL USA Animal Science, School of Agricultural Sciences, Southern Illinois University, Carbondale, IL, USA

**Keywords:** Tributyltin, toxicity, placenta, offspring, ovary, testes

## Abstract

Tributyltin (TBT) is an organotin compound and a common persistent environmental pollutant with endocrine-disrupting chemical (EDC) actions. It can accumulate in the environment at various concentrations throughout the food chain in the ecosystem, posing a risk to human health, especially during critical periods such as gestation and fetal and offspring development. In this review, we report the results of studies describing the consequences of TBT exposure on placental and reproductive parameters in offspring of both sexes. Results from *in vivo* and *in vitro* studies clearly indicate that TBT causes adverse effects on placental development and reproductive parameters in offspring. However, substantial knowledge gaps remain in the literature, requiring further research to better understand the mechanisms behind TBT effects on placental and reproductive disruption in offspring.

## INTRODUCTION

**Figure 1:**
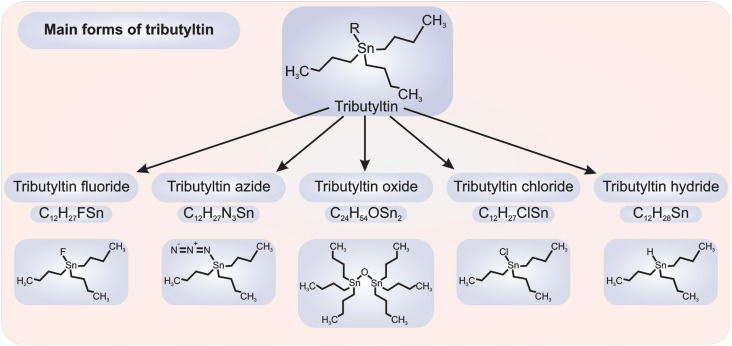
Five of the most common tributyltin chemicals.

Tributyltin (TBT) is a manmade organometallic chemical that belongs to the family of organotin compounds (1,2). Organotin compounds are synthetic chemical tetravalent derivatives of tin (IV), with a general formula of R(4-n)SnXn, where R represents organic substituents and X can be a halide, anion, or an organic group linked covalently through a heteroatom (*e.g.*, O, N, S, Cl) (2,3). The most common forms of TBT, *i.e.*, (C_4_H_9_)_3_Sn^+^, feature fluoride, oxide, chloride, azide, or hydride as the negatively charged X group (Figure 1).

**Figure 2:**
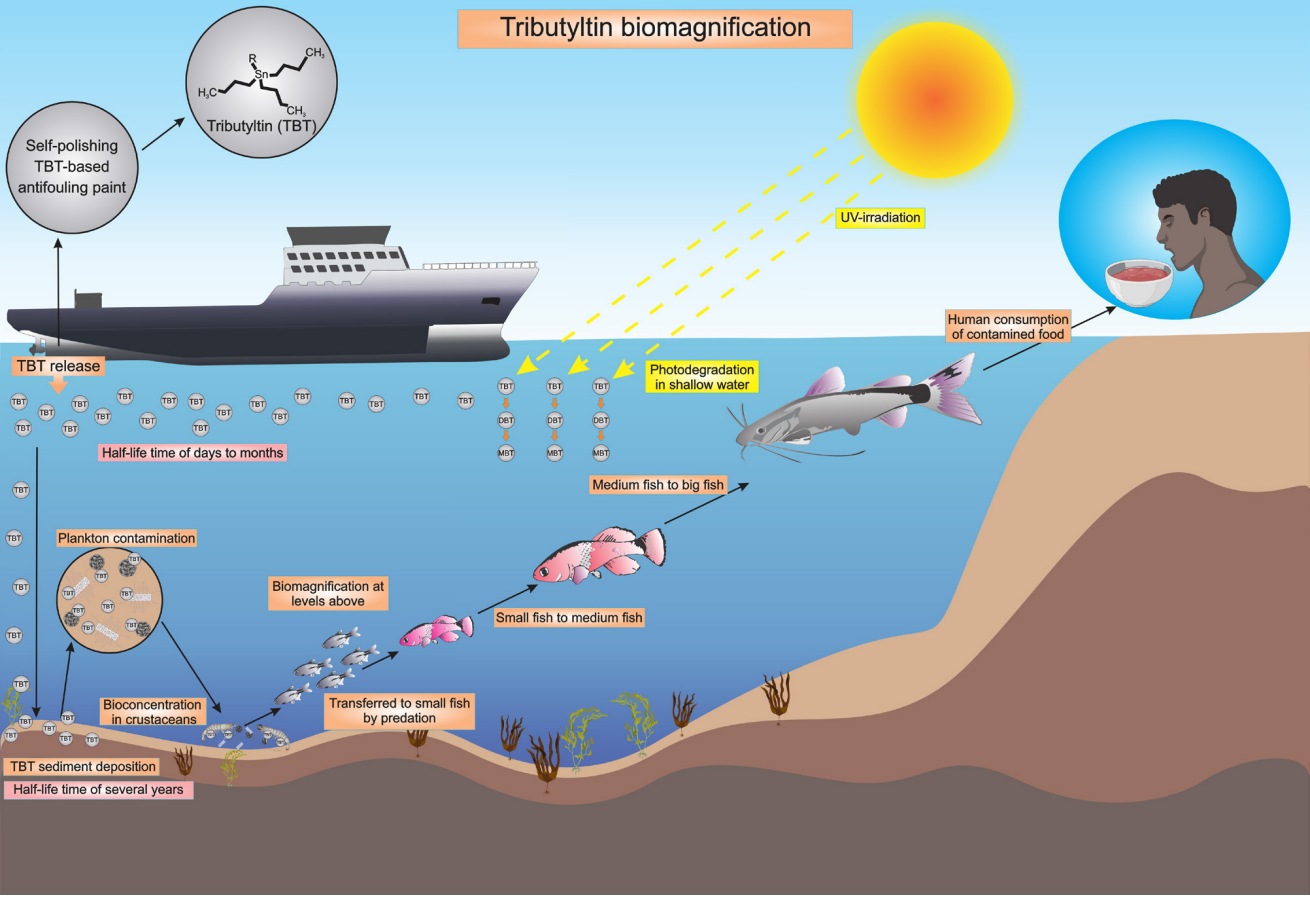
Pathways of tributyltin (TBT) accumulation in the environment.

The most studied organotin compound is TBT due to its toxicity and widespread industrial use, such as in the manufacturing of antifouling paints, as a preservative in woods, papers, and textiles, as a component in broad-spectrum biocides (*e.g*., agricultural fungicides), and as a stabilizer in plastic production (4,5). The environment is widely contaminated by TBT and its metabolites, such as dibutyltin (DBT) and monobutyltin (MBT), due in part to inappropriate disposal of products that contain TBT, such as paints and pesticides. Due to its lipophilic properties and persistence, TBT is able to bioaccumulate and biomagnify in the food chain, leading to ecosystem-level contamination and health impacts on wildlife and humans (1,6). Notably, TBT has a degradation half-life of days to months in water and up to several years in sediment (7). Experiments have revealed that equilibrium partitioning predictors, such as the octanol-water partitioning coefficient, are not good predictors of TBT partitioning and that the environmental fate of TBT is dependent on the environmental condition (8) (Figure 2).

The use of products containing TBT has increased worldwide contamination, leading to deleterious consequences of TBT exposure across ecosystems (5,9). Due to TBT toxicity in target and nontarget species, the International Marine Organization (IMO), through the International Convention on the Control of Harmful Antifouling Systems on Ships, banned the use of TBT-based antifouling paints (2008), and the Rotterdam Convention (RC; www.pic.int) forbade the sale of TBT (10,11). As a result of these efforts, a reduction in TBT concentrations and biological effects has been observed in coastal areas worldwide (12,13). However, TBT-based paints were revealed to be illegally manufactured in the USA in 2014 and sold throughout the Caribbean and Central America (14,15). Recent evidence suggests that these practices have continued and that they may be taking place over a much wider geographical region, as fresh inputs of TBT continue to be reported worldwide (15-22).

In humans, the principal route of exposure to TBT and its metabolites occurs by ingestion of contaminated water, beverages, and food, particularly seafood (23,24). The European Food Safety Authority (EFSA) and the United States Environmental Protection Agency (EPA) established a human tolerable daily intake (TDI) of TBT of 250 and 300 ng/kg/day, respectively. The TDI was derived by applying a 100-fold safety factor from the no-observed-adverse-effect level of 25 μg/kg/day in mice (25,26). Concentrations of TBT in human blood have been measured at approximately 155 ng/mL in a study from Michigan, US (27), while TBT assessment in human placenta samples collected in Finland between 1997 and 1999 found a level of 0.32 ng/g (28). In 2018, MBT (∼6 ng/g), DBT (∼6 ng/g), TBT (∼1 ng/g), and tetrabutyltin (TeBT, ∼5 ng/g) were detected in human urine from adults in Iowa, US (29). Epidemiological TBT studies have found TBT concentration in human tissue between 0.01 and 85.0 ng/g (28,30-32).

Studies have evaluated inorganic tin concentrations resulting from the metabolism of TBT or other organotin compounds (33,34). Inorganic tin is poorly absorbed in the gastrointestinal tract of mammals and could be present from the cellular metabolism of organotins, *i.e.*, TBT>DBT>MBT>inorganic tin (35,36). Thus, it has been suggested that an important fraction of tin may be present in the form of inorganic tin in mammalians’ bodies as a result of organotin contamination (33,37). Podratz and cols. (2015) identified an increase in serum tin concentration (∼40 ng/g) as a result of exposure to seafood contaminated with organotin in rats. Similarly, exposure of rats to TBT at a dose of 100 ng/kg/day for 15 days was associated with increased serum and ovarian tin concentration (∼49 and 579 ng/g, respectively) (34). Evaluation of human urine from adults in Iowa, US, in 2018, reported a range of tin concentration between 0.07 ng/mL and 2.10 ng/mL (29). This tin evaluation could serve as a new tool for assessing organotin contamination in the human body.

Exposure to TBT results in complex deleterious consequences, involving different molecular pathways and impairing several physiological functions (1,38,39). In the endocrine system, TBT acts as an endocrine-disrupting chemical (EDC) and metabolic and reproductive toxicant (3,40). According to the Endocrine Society and the World Health Organization (WHO), EDCs are "an exogenous substance or mixture that alters function(s) of the endocrine system and consequently causes adverse health effects in an intact organism, or its progeny, or (sub)populations" (41,42). The structural similarities between EDCs and physiological hormones lead to EDCs being able to interfere with hormone receptor actions (40,43). Thus, a major mechanism of action of EDCs is their interaction with receptors for steroid hormones, thyroid receptors, peroxisome proliferator-activated receptors (PPARs), and aryl hydrocarbon receptors (AhR) (44). Notably, EDCs bind to nuclear receptors to act as agonists or antagonists, leading to (A) blocking of the action of natural hormones via their union with a binding site for endogenous hormones and/or (B) increased/decreased expression of downregulated genes and/or (C) changes in genomic and nongenomic nuclear receptor pathways, nonsteroidal receptors, and ion channels leading to abnormal molecular pathway, enzyme activity/expression, and transcriptional coactivation and or repressors (40,45). Furthermore, EDCs bind to plasma-transporting proteins, thereby disturbing hormonal transport, tissue distribution, breakdown, and activity in the bloodstream (44). Recent studies have also reported that EDCs are able to change epigenetic regulation and alter genomic methylation, causing histone and metabolomic modifications, often leading to deleterious transgenerational consequences (46,47).

Thus, the purpose of this review is to discuss recent research on the impact of TBT on the placenta and its reproductive consequences in offspring. We focus on *in vitro* and *in vivo* studies of TBT exposure during fetal development from both human and animal models. Furthermore, we review the effects of TBT on placental morphophysiology and reproductive offspring consequences (ovary and testes).

### Main toxicological mechanisms of TBT

**Figure 3:**
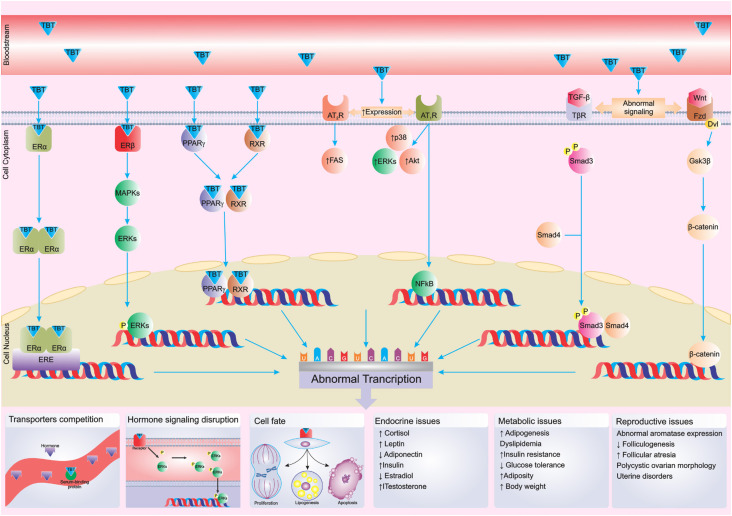
Summary of main tributyltin (TBT) mechanisms in the endocrine system.

Specifically, TBT acts through several complex toxicological molecular mechanisms to impair endocrine physiology (48-51) (Figure 3). It is able to bind to both estrogen receptor (ER) subtypes – ER-alpha (Erα) and ER-beta (ERβ) – in adipocytes, leading to disruption of estrogenic action depending on dose and cell type (48). The estrogenic actions of TBT involve both classical and nonclassical membrane-mediated pathways, primarily through activation of the mitogen-activated protein kinase (MAPK) pathway, which includes the phosphorylation of extracellular signal-regulated kinases (ERKs) (50). In addition, TBT changes aromatase expression and activity, which can alter estrogen actions (50,52). Cyclic adenosine 3’,5’-monophosphate (cAMP) response element-binding protein (CREB) is phosphorylated most likely due to TBT-mediated activation of MAPK, upregulating CRE-dependent transactivation (50). In varying doses, TBT can inhibit the activity of 11β-hydroxysteroid dehydrogenase type 2 (11β-HSD2), which is responsible for the inactivation of cortisol and could increase glucocorticoid levels (53). Furthermore, TBT acts as a potent nanomolar activator of both retinoid X receptors (RXRs) and PPARγ, leading to obesogenic actions and impairing metabolic function (49,54). In the mesenchymal stem cell, TBT activates RXR signaling, leading to altered expression of enhancer of zeste homolog 2 (EZH2), modified expression of genome-wide histone 3 lysine 27 trimethylation (H3K27me3), and reprogramming stem cells to increase adipogenesis (55). Additionally, TBT modulates angiotensin-II receptor proteins (AT1R and AT2R), the nuclear factor-κB (NF-κB) signaling pathway, and serine/threonine protein kinase (AKT) and ERK expression in abnormal adipocytes, resulting in features of metabolic syndrome (56). A recent study showed that TBT affects wingless-related integration site (Wnt) and transforming growth factor-β/small mothers against decapentaplegic (TGF-β/Smad) signaling, leading to uterine disorders (57) (Figure 3).

## MATERIAL AND METHODS

The articles used in this current review were selected from the PubMed database without exclusion based on publication year of study or positive or negative findings. We utilized the following inclusion criteria: work evaluating direct TBT exposure effects on the placenta or offspring ovary and testes using *in vitro* and *in vivo* mammalian models. We included searches on any TBT dose, time, and life stage of exposure. Only data that described the relationship between relevant TBT dose or exposure and effect were included in the review. Each original study was critically evaluated for appropriateness of the journal indexing, model, use of adequate controls (negative and positive), range of doses tested, methodology, and statistical methods. In addition, data analyzing the combined effect of more than one type of EDC and TBT were not included. Conference proceedings and articles not written in English were excluded. To the extent possible, we made efforts to be consistent in the description, discussion, and integration of findings on the effects of TBT exposure on the placenta and offspring reproductive consequences in both sexes.

### TBT and placental consequences

During pregnancy, the placenta plays an important role in organogenesis and tissue differentiation (58,59). Previous studies have reported that EDC exposures during gestation can cause adverse outcomes in exposed children, such as thalidomide leading to limb malformations and methylmercury causing Minamata disease (60). Embryogenesis is a critical window of development, characterized by heightened sensitivity to environmental factors that may interfere with the fetal reprogramming process. Thus, the developmental origins of health and disease (DOHaD) hypothesis posits that an adverse environment experienced during development can increase the risk of disease later in life (61-63). Human and other mammalian experimental pregnancy models have reported abnormalities as a result of exposure to synthetic hormones, and inadvertent exposures to environmental chemicals, including EDCs such as TBT (64-66).

Recent studies have shown that TBT exposure during early pregnancy can cause many adverse effects on placental function. In one study, pregnant mice were fed 0, 0.2, and 2 mg/kg/day of TBT from gestational day (GD) 1 to 8 or 13 (67). Exposure to TBT led to an increased rate of resorbed embryos and reduced fetal weight of embryos that were able to develop at GD 13. Moreover, placental weight and area were significantly reduced. In the same study, further tests showed that the laminin immunoreactivity was reduced in the 2 mg/kg/day group at GD 13. These changes could be linked to irregular implantation and placentation in pregnant mice exposed to TBT. The study also found that the placenta-to-body weight ratio was reduced in the 0.2 mg/kg/day and 2.0 mg/kg/dayTBT exposures. Exposure to TBT was associated with atrophy of the ectoplacental cone at GD 8. The placental areas of the spongiotrophoblast and labyrinth were significantly reduced in mice exposed to 2.0 mg/kg/day TBT compared with those exposed to control conditions, showing abnormal placentation. Moreover, the spongiotrophoblast-to-labyrinth ratio in the 2.0 mg/kg/day TBT exposure was found to be increased, along with the expression of the placental development-related molecules fos-related antigen-1 (FRA1), eomesodermin (EOMES), heart and neural crest derivatives expressed 1 (HAND1) and achaete-scute family BHLH transcription factor 2 (ASCL2). The results also showed an inhibition of placental proliferation and induced upregulation of p53 and cleaved caspase-3 (apoptosis marker) and reduced levels of the Bcl-2 protein. Furthermore, TBT was found to induce thymocyte apoptosis through oxidative stress. The study also indicated that TBT exposure during gestation induced the upregulation of markers of oxidative stress alongside declining levels of antioxidant enzymes catalase and superoxide dismutase in the mouse placenta, which was observed on GD 13 (67). These placental abnormalities related to irregular gene expression, oxidative stress, and apoptosis could contribute to improper fetal development.

Exposure to TBT is able to change placental steroidogenesis. The human placental choriocarcinoma cell line JAr was cultured and treated with various concentrations of TBT and its metabolites for 48 hours to investigate their effects on the catalytic activity and mRNA expression of 17β-hydroxysteroid dehydrogenase type 1 (17β-HSD1). The results showed that mRNA expression and enzyme activity of 17β-HSD1 increased after exposure to TeBT and tributylvinyltin. Moreover, the metabolites of TBT, DBT, and MBT caused 17β-HSD1 to enhance its activity without increasing mRNA expression. This result suggests that organotin compounds are stimulators of 17β-estradiol biosynthesis by potentially promoting estrogenic action to enhance 17β-HSD1 activity in the human placenta *in vitro* (68).

To assess the impacts of TBT during pregnancy, timed-pregnant rats were gavaged with TBTCl (0, 0.25, 2.5, 10, or 20 mg/kg) from GD 0 to 19 or GD 8 to 19. The dams were sacrificed on GD 20. Gas chromatography measured TBT and its metabolite DBT in the blood at almost equal concentrations. Maternal weight gain was reduced due to TBTCl administration and mean placental weights were increased in the groups exposed to 10 mg/kg of TBTCl from GD 0 to 19 or GD 8 to 19. Although placental enlargement was present, reductions in fetal weights in litters were also observed at the highest dose of 20 mg/kg. This indicates that any enlargement in the placenta enhanced by TBTCl exposure is not associated with induced fetal growth. Instead, it shows its correlation with reduced fetal weights and embryo or fetal loss. Postimplantation losses were observed at the high TBT dose of 20 mg/kg. Lost embryos occurred in litters (three) from the dams exposed to the highest dose (69).

In another study investigating the effect of DBT on rat placentas, 85 pregnant rats were assigned to three groups of 23-26 rats each. Dam weights were recorded on GDs 6-21. The pregnant rats were sacrificed on GD 9.5, 11, 13, 14, 15, 17, and 21. Additionally, DBT was orally dosed at 20 mg/kg during days 7-9 of gestation (GD 7-9 group) and 20 mg/kg during days 10-12 of gestation (GD 10-12 group). No histological changes were observed in the GD 7-9 group in the decidual mass, metrial gland, and ectoplacental cone compared with the control group. Apoptosis was observed in the labyrinth zone during GD 11-21, showing abnormal placentation. Exposure to DBT during GD 0-3 also reduced progesterone levels, which induced failure in embryo implantation (70). In the GD 10-12 treatment group, some placentas on GDs 17 and 21 showed multiple cysts consisting of cytolysis and residual glycogen cell islands. These lesions are speculated to be induced by exposure to DBT and are considered retardation in the fetal parts of the placenta. In addition, basal zone thickness decreased from GD 11 to GD 15 in the GD 10-12 treatment group, while DBT induced apoptosis and/or mitotic inhibition in the labyrinth zone in the placenta, which can lead to the induction of intrauterine growth retardation (70).

Overall, the mechanisms through which TBT and its metabolites affect placental and fetal development need further exploration in future studies. There is growing evidence of direct disruption by TBT. Current studies suggest TBT may alter placental function on a cellular level by either inhibiting the labyrinth zone (4) and/or promoting estrogenic activity in the human placenta (68). Thus, TBT exposure could be associated with abnormal embryo implantation and placentation that may contribute to abnormal fetal development or lead to future dysfunction, such as reproductive abnormalities.

### TBT and reproductive offspring consequences

Important studies have investigated how exposure to TBT during gestation and/or lactation can lead to reproductive abnormalities in female and male offspring, including notable observations of sex differences (Table 1).

**Table 1:** Effects of tributyltin (TBT) on reproductive parameters in offspring

Parameters	Animal model			
	Pregnant Rat (5, 25, and 125 µg/kg)	Pregnant rat (2 mg/kg)	Pregnant mouse (1, 10, and 100 µg/kg)	Pregnant Rat (0.25-20 and 1-10 mg/kg)
**Female offspring**				
Body weight	↓	↓	NA	NR
Vaginal opening	↓	↔	NA	NR
Estrous cyclicity	Impaired	↔	NA	NR
Ovarian weight	NR	↓	NA	NR
Ovarian morphology	NR	NR	NA	Impaired
Ovarian cell apoptosis	NR	NR	NR	↑
Ovarian gene expression	NR	NR	NR	↓ Itgb1, Arf5, Hsp90ab1
Gonadotropin levels	NR	↑ FSH, ↔LH	NA	NR
Steroid levels	NR	↔E2	NA	NR
**Male offspring**				
Body weight	↓ F1 / F2	NR	NR	NR
Cryptorchidism	↔	↑	↑	NR
Preputial separation	NR	↔	NR	NR
Testicular weight	↓ F1	↔	↔	NR
Testicular morphology	↔	↔	Impaired, ↔	Impaired
Testicular cell apoptosis	NR	NR	NR	NR
Testicular gene expression	NR	NR	NR	↑ Itgb1, Tnfrsf1b, Lhcgr, oxidative stress-related genes
Sperm concentration	↓ F1 / F2	↔	↓	NR
Sperm motility	↓ F1 / F2	↔	↓	NR
Gonadotropin levels	↔LH F1, ↑F2	↔ FSH, LH	NR	NR
Steroid levels	↑T F1, ↔F2; ↓E2 F1 / F2	↔T	↔, E2 T	NR
References	[70,71]	[73,74]	[72,75]	[77,78]

Note:

↓ = decreased,

↑ = increased,

↔ = similar.

Abbreviations: *Arf5*, ADP-ribosylation factor 5; E2, estrogen; F1, first generation; F2, second generation; FSH, follicle-stimulating hormone; *Hsp90ab1*, heat shock 90 kDa protein 1 beta; *Itgb1*, integrin beta 1; LH, luteinizing hormone; *Lhcgr*, luteinizing hormone/choriogonadotropin receptor; NA, not applicable; NR: Not reported; T, testosterone; *Tnfrsf1b*, TNF receptor superfamily member 1b.

Both F1 and F2 male rat offspring of dams who ingested 5, 25, or 125 μg of TBTCl per g of diet (5, 25, and 125 ppm TBTCl diet, respectively) from gestation to lactation showed male reproductive complications (Table 1) (71). Specifically, the body weight of the 125 ppm TBTCl pups was reduced on postnatal day (PND) 1 and was consistently reduced during the lactation in both the F1 and F2 generations. No significant changes were observed on the day of testes descended into the scrotum for control and TBT groups in both F1 and F2 generations. However, the weights of F1 testes were reduced in all TBT male offspring groups compared with the control group. Both spermatids and testes and sperm/caudal concentrations were reduced in both F1 and F2 generations in the 125 ppm TBTCl dose group. Other sperm parameters, such as sperm motility and percentage of abnormal sperm, were similar between control and TBT groups in both F1 and F2 generations, except for an increase in absent tail sperm in the F1 generation of the 125 ppm TBTCl group. Histopathologic changes in the seminiferous tubules of testes were occasionally observed in the rats of the TBTCl groups in the F1 generation, including vacuolization of seminiferous epithelium, spermatid retention in the epithelium, delayed spermiation, and germ cell degeneration. However, the frequencies were low, and the histopathologic findings of these rats are not likely abnormal. There was a dose-dependent increase in serum testosterone concentration in the rats fed the TBTCl diets in the F1 generation, but testosterone concentration did not increase in the F2 generation. The concentration of luteinizing hormone (LH) in the TBTCl-treated rats did not increase in the F1 generation. However, LH level increased in a dose-dependent manner in the F2 generation. In addition, estrogen concentration decreased in F1 and F2 male pups of dam rats fed the 125 ppm TBTCl diet (71).

Using a similar experimental model of TBT exposure during pregnancy, Ogata and cols. (2001) reported that F1 and F2 female offspring with a whole-life dietary concentration of 125 ppm of TBTCl showed a delay of approximately 6 days for the vaginal opening, suggesting an irregular pubertal onset that could contribute to impaired regular estrous cyclicity (Table 1) (72). In mice exposed to TBTCl 10 or 100 µg/kg of body weight/day from GD 6 of pregnancy through lactation, the female offspring showed both an early vaginal opening and an early first day of estrus, indicating features of early puberty (73). In the same study, these female offspring showed no alteration in the weight of the female sex organs or sex hormone levels. However, they presented irregularities in the estrous cyclicity, spending more time in the estrus and diestrus phases compared with control animals (73). In addition, female offspring of dam rats who ingested 25 ppm of TBT (approximately 2 mg/kg) during the perinatal period from gestation to lactation showed a reduction in body weight at PND 28, 56, and 84 and a reduction in ovary weight at PND 90 (Table 1). An increase in serum follicle-stimulating hormone (FSH) concentration was noted in perinatally exposed female pups. No significant changes were observed in the day of vaginal opening or serum estrogen and LH concentrations in perinatally exposed female pups compared with controls (74). Using the same TBT exposure model to assess male pups, Makita (2008) (74) did not observe significant changes in the male reproductive system, including preputial separation day, testes’ weight, sperm concentration, motility and abnormalities, and serum testosterone, FSH and LH concentrations (75). Thus, in this rat model, female offspring exposed to TBT during pregnancy presented important reproductive abnormalities compared with male offspring, suggesting females would be more susceptive to reproductive abnormalities induced by TBT exposure.

Pregnant mice were administered TBTCl 0, 1, 10, or 100 µg/kg/day from GD 6 through lactation, showing male offspring reproductive complications (76). Specifically, TBTCl decreased sperm counts and motility on PNDs 49 and 152 in male offspring. In addition, an increase in sperm abnormalities was observed in exposed offspring on PND 49. Histopathological examination of offspring testes showed a dose-dependent increase in germ cells sloughing in the seminiferous tubules. Mouse pups from dams treated with 10 µg TBTCl/kg exhibited decreased intratesticular estrogen concentration on PND 49. No significant differences in serum estrogen, testosterone levels, or intratesticular testosterone levels were detectable between control and TBTCl-exposed offspring (76). In another study using the same pregnancy model, TBTCl 100 µg/kg/day retarded testes descent in offspring (73). Recently, Shioda and cols. (2022) evaluated the transcriptomic impact of F0 exposure to TBT on testicular cells in offspring. Interestingly, these authors did not detect significant global transcriptomic effects on testicular cells of F1 or F3 male offspring, except in the somatic cell population of F1 fetal testes (77). Thus, these results suggest that perinatal exposure of F1 males to TBT affects testicular somatic cell expression of transcription factors that may be involved in mesenchymal lineage differentiation to muscles or adipocytes, although such effects did not persist in the F3 testes.

Pregnant rats were gavaged with TBT (0.25, 2.5, 10, or 20 mg/kg) from GD 0 to 19 or 8 to 19. On GD 20, fetal testes and ovaries were evaluated (Table 1) (78). At the highest TBT doses (10 or 20 mg/kg), the number of Sertoli cells and gonocytes was reduced, there were large intracellular spaces between Sertoli cells and gonocytes, and there was an increased abundance of lipid droplets in the Sertoli cells. An electron microscopy evaluation revealed abnormal dilated endoplasmic reticulum in Sertoli cells and gonocytes. In the ovaries, TBT (20 mg/kg, GD 0-19; 10 mg/kg, GD 8-19) reduced the number of germ cells by 44% and 46%, respectively. Both experimental groups (GD 0-19 and 8-19) showed a trend toward increased numbers of apoptotic cells with higher doses of TBT. Ovaries exposed to 10 mg/kg of TBT (GD 0-19) had increased numbers of apoptotic cells compared with those of controls. Examining gene expression profiles in the testis revealed that, compared with control, 40 genes out of 1,176 tested were upregulated more than twofold, including the integrin beta 1 (*Itgb1*), tumor necrosis factor receptor superfamily member 1A (*Tnfrsf1a*), activating transcription factor 2 (*Atf2*), and inhibitor of DNA binding 2 (*Id2*), as well as the oxidative stress-related genes cytochrome P450, subfamily 51 (*Cyp51a1*), superoxide dismutase 1 (*Sod1*), cytochrome c oxidase subunit 1 (*Mt-co1*), ATP synthase, H+ transporting, mitochondrial F1 complex, beta polypeptide (*Atp5f1b*), ATP synthase, H+ transporting, mitochondrial F0 complex, subunit b, isoform 1 (*Atp5pb*), insulin-like growth factor 2 receptor (*Igf2r*), and luteinizing hormone/choriogonadotropin receptor (*Lhcgr*). While no genes were upregulated in the TBT-exposed fetal ovaries, eight genes were downregulated, including the integrin beta 1 (*Itgb1*), cell division cycle 25 homolog B (*Cdc25b*), heat shock 90 kDa protein 1 beta (*Hsp90ab1*), mitogen-activated protein kinase kinase 2 (*Map2k2*), adenylate cyclase 2 (*Adcy2*), and ADP-ribosylation factor 5 (*Arf5*), among others (Table 1). Furthermore, male rats exposed to TBTCl 10 mg/kg in GD 15 showed increased expression of LH β-subunit mRNA in the male pituitary without significant changes in testicular steroidogenic acute-regulatory protein (StAR) mRNA expression compared with control rats (79). Thus, in this study, TBT exposure in pregnant mice led to intense changes in gene expression in the tests compared with the ovaries of offspring (78).

### Limitations and strengths

This review reveals a number of limitations in the available body of research on TBT exposure and placental and reproductive complications in offspring. For the *in vitro* studies, many experiments used doses that do not reflect real human exposure. In addition, these studies evaluated types of cells individually (*e.g.*, adipocytes, human breast cancer cells, stem cells, human placental cells), which may eliminate or distort signaling and microenvironment structure differences. For the *in vivo* studies, rodents are the only model used to date, and the inclusion of other mammalian species could improve the translational relevance to humans. The *in vivo* studies described in this review have focused on TBT effects in experimental models. Few studies have evaluated the effects of TBT in human samples, such as placenta, blood, or gonads. Humans are exposed to low doses over a long period, but low-dose and chronic exposure studies are regretfully lacking. Furthermore, *in vivo* studies analyzing preconception exposure or paternal exposures and new reproductive offspring complications are limited. Another important point is that few studies reported an interaction between TBT and other EDCs, including mixtures and their consequences or interaction with an unbalanced diet (80). It is equally important to consider that TBT, as well as other EDCs, affect thyroid function. Even though the male gonad was considered to be unresponsive to thyroid hormones in the 1950s, it is quite established today that thyroid hormone receptors are present in Sertoli cells, where their expression changes during gonadal development, being highly expressed in the fetus and the early postnatal life. They are expressed in gonadotrophs too, as shown by alterations in FSH and LH levels in hyperthyroidism and hypothyroidism. Moreover, thyroid hormones are essential for the general development of the central nervous system, and in the initial phase of fetal development, they are provided to the fetus from the mother, transported by the placenta, through specific transport proteins (MCT8), which can also be affected by EDCs (81,82). These gaps leave room for further study for an effective understanding of the damage that TBT can cause to placental and gonadal offspring development, maturation, and function.

The main strength of this review is that, to our knowledge, it covers all studies published on the morphophysiological and molecular effects of TBT on the placenta and reproductive parameters of offspring to provide an integrated overview of the state of the science. Further, this review emphasizes areas for future study.

This review reveals some important gaps in the literature that need to be filled to effectively assess the effects of TBT on the placenta and reproductive parameters of offspring, and the biological mechanisms involved. Here, we describe these gaps. The majority of *in vitro* studies have been carried out with commercial cell lines, which may not be consistent with the real specificities of placental or gonadal cells. In addition, most *in vitro* studies use cells of unknown sex. Thus, future *in vitro* studies should develop cell lines more consistent with the characteristics (including the microenvironment) of placental and gonadal cells and develop advanced models for the same purpose. In addition, sex-specific analyses should be performed, and doses should be used to mimic human exposure. Some options for alternative *in vitro* models, such as the use of chips and 3D models, are already available (83,84) and have been described in a recent review (85). Further development of organoid models of the placenta would improve the translational potential of cell culture models.

As discussed above, *in vivo* studies in rodents are significantly lacking in dose amount and method of exposure that are relevant to human exposure. In addition, studies on other mammals with translational application to humans are needed. A recent review of animal models of the placenta describes the advantages and limitations of models, such as guinea pigs, sheep, and nonhuman primates, and points out the need for studies to focus on temporality throughout pregnancy, complications with pregnancies, hormonal aspects related to the placenta, and other particularities that must be considered when choosing a study animal for human translation (86,87). Furthermore, the results of animal studies should be compared to those of human epidemiology studies and human *in vitro* models to assess their translational value.

In human studies, we observe a need for more studies analyzing preconception exposures to TBT, paternal exposure to TBT, and exposure throughout the entire pregnancy. Additionally, such studies should focus on measuring TBT levels in urine across all trimesters and throughout the entire placenta, as well as assessing placental endpoints at various stages of pregnancy, and evaluating TBT in the umbilical cord and offspring blood, among other assessments. Every study should also observe differences between the placental sexes. The preconception period is also relevant and needs to be studied more deeply, along with paternal exposure to TBT. In addition to all the above factors, the literature would be enriched by more studies with larger sample sizes.

In conclusion, increasing concern regarding the adverse effects of TBT on gestation and fetal heath has driven research investigating the potential biological mechanisms of action and physiological damages behind TBT exposure. This review compiles studies that enhance the understanding of TBT exposure effects in the context of placental dysfunction and reproductive issues affecting the offspring. Despite gaps and contradictory data in the literature, the studies described herein strongly suggest that exposure to TBT leads to impaired placental function and abnormal reproductive features in offspring of both sexes. The biological mechanisms, exposure timing and dose, and the specific translation to human fetal, gonadal, and gestational health are poorly understood. Future molecular studies will be key to improving our understanding of these effects. Thus, additional research is needed to understand how environmental exposure to TBT can drive human placental and offspring reproductive disruption. Specifically, well-designed animal studies and human studies are necessary to understand how TBT and its metabolites, as well as other EDCs, interact with placental and gonadal receptors to disrupt downstream molecular signaling and how TBT can change the placental and reproductive developmental microenvironment to lead to abnormal function.

## References

[1] Graceli JB, Dettogni RS, Merlo E, Niño O, da Costa CS, Zanol JF (2020). The impact of endocrine-disrupting chemical exposure in the mammalian hypothalamic-pituitary axis. Mol Cell Endocrinol.

[2] Hoch M, A. Press (2001). The Rat Brain in Stereotaxic Coordinates. Applied Geochemistry.

[3] Barbosa KL, Dettogni RS, da Costa CS, Gastal EL, Raetzman LT, Flaws JA (2022). Tributyltin and the female hypothalamic-pituitary-gonadal disruption. Toxicol Sci.

[4] Antizar-Ladislao B (2008). Environmental levels, toxicity and human exposure to tributyltin (TBT)-contaminated marine environment. A review. Environ Int.

[5] Fent K (1996). Ecotoxicology of organotin compounds. Crit Rev Toxicol.

[6] Podratz PL, Merlo E, de Araújo JFP, Ayub JGM, Pereira AFZ, Freitas-Lima LC (2020). Disruption of fertility, placenta, pregnancy outcome, and multigenerational inheritance of hepatic steatosis by organotin exposure from contaminated seafood in rats. Sci Total Environ.

[7] European Chemical Agency (ECHA) (2004). Agreement of the member state committee on identification of bis(tributyltin) oxide (TBTO) as a substance of very high concern. Regulation (EC) No 1907/2006.

[8] Meador JP (2000). Predicting the fate and effects of tributyltin in marine systems. Rev Environ Contam Toxicol.

[9] Graceli JB, Sena GC, Lopes PF, Zamprogno GC, da Costa MB, Godoi AF (2013). Organotins: A review of their reproductive toxicity, biochemistry, and environmental fate. Reprod Toxicol.

[10] Maciel DC, Castro ÍB, de Souza JRB, Yogui GT, Fillmann G, Zanardi-Lamardo E (2018). Assessment of organotins and imposex in two estuaries of the northeastern Brazilian coast. Mar Pollut Bull.

[11] International Maritime (2009). Organization. Guidance on best management practices for removal of anti-fouling coatings from ships, including TBT hull paints.

[12] Lahbib Y, Abidli S, Trigui-El Menif N (2018). First assessment of the effectiveness of the international convention on the control of harmful anti-fouling systems on ships in Tunisia using imposex in Hexaplex trunculus as biomarker. Mar Pollut Bull.

[13] Laranjeiro F, Sánchez-Marín P, Oliveira IB, Galante-Oliveira S, Barroso C (2018). Fifteen years of imposex and tributyltin pollution monitoring along the Portuguese coast. Environ Pollut.

[14] Turner A, Glegg G (2014). TBT-based antifouling paints remain on sale. Mar Pollut Bull.

[15] Uc-Peraza RG, Castro ÍB, Fillmann G (2022). An absurd scenario in 2021: Banned TBT-based antifouling products still available on the market. Sci Total Environ.

[16] Batista-Andrade JA, Caldas SS, Batista RM, Castro IB, Fillmann G, Primel EG (2018). From TBT to booster biocides: Levels and impacts of antifouling along coastal areas of Panama. Environ Pollut.

[17] Mattos Y, Stotz WB, Romero MS, Bravo M, Fillmann G, Castro ÍB (2017). Butyltin contamination in Northern Chilean coast: Is there a potential risk for consumers?. Sci Total Environ.

[18] Castro ÍB, Iannacone J, Santos S, Fillmann G (2018). TBT is still a matter of concern in Peru. Chemosphere.

[19] Kim T, Jeon S, Hong S, Song SJ, Kwon BO, Ryu J (2017). Spatiotemporal distributions of butyltin compounds in various intertidal organisms along the Samcheok and Tongyeong coasts of Korea. Chemosphere.

[20] Lam NH, Jeong HH, Kang SD, Kim DJ, Ju MJ, Horiguchi T (2017). Organotins and new antifouling biocides in water and sediments from three Korean Special Management Sea Areas following ten years of tributyltin regulation: Contamination profiles and risk assessment. Mar Pollut Bull.

[21] Romanelli G, Berto D, Calace N, Amici M, Maltese S, Formalewicz M (2019). Ballast water management system: Assessment of chemical quality status of several ports in Adriatic Sea. Mar Pollut Bull.

[22] Wang X, Kong L, Cheng J, Zhao D, Chen H, Sun R (2019). Distribution of butyltins at dredged material dumping sites around the coast of China and the potential ecological risk. Mar Pollut Bull.

[23] Azenha M, Vasconcelos MT (2002). Butyltin compounds in Portuguese wines. J Agric Food Chem.

[24] He S, Li P, Li ZH (2021). Review on endocrine disrupting toxicity of triphenyltin from the perspective of species evolution: Aquatic, amphibious and mammalian. Chemosphere.

[25] (2004). Opinion of the Scientific Panel on contaminants in the food chain [CONTAM] to assess the health risks to consumers associated with exposure to organotins in foodstuffs. EFSA J.

[26] Vos JG, De Klerk A, Krajnc EI, Van Loveren H, Rozing J (1990). Immunotoxicity of Bis(tri-n-butyltin)oxide in the rat: Effects on thymus-dependent immunity and on nonspecific resistance following long-term exposure in young versus aged rats. Toxicol Appl Pharmacol.

[27] Kannan K, Senthilkumar K, Giesy JP (1999). Occurrence of Butyltin Compounds in Human Blood. Environ Sci Technol.

[28] Rantakokko P, Main KM, Wohlfart-Veje C, Kiviranta H, Airaksinen R, Vartiainen T (2014). Association of placenta organotin concentrations with growth and ponderal index in 110 newborn boys from Finland during the first 18 months of life: A cohort study. Environ Health.

[29] Gadogbe M, Bao W, Wels BR, Dai SY, Santillan DA, Santillan MK (2019). Levels of tin and organotin compounds in human urine samples from Iowa, United States. J Environ Sci Health A Tox Hazard Subst Environ Eng.

[30] Kannan K, Corsolini S, Focardi S, Tanabe S, Tatsukawa R (1996). Accumulation pattern of butyltin compounds in dolphin, tuna, and shark collected from Italian coastal waters. Arch Environ Contam Toxicol.

[31] Nielsen JB, Strand J (2002). Butyltin compounds in human liver. Environ Res.

[32] Rantakokko P, Main KM, Wohlfart-Veje C, Kiviranta H, Airaksinen R, Vartiainen T (2013). Association of placenta organotin concentrations with congenital cryptorchidism and reproductive hormone levels in 280 newborn boys from Denmark and Finland. Hum Reprod.

[33] Dorneles PR, Lailson-Brito J, Fernandez MA, Vidal LG, Barbosa LA, Azevedo AF (2008). Evaluation of cetacean exposure to organotin compounds in Brazilian waters through hepatic total tin concentrations. Environ Pollut.

[34] Podratz PL, Merlo E, Sena GC, Morozesk M, Bonomo MM, Matsumoto ST (2015). Accumulation of organotins in seafood leads to reproductive tract abnormalities in female rats. Reprod Toxicol.

[35] Krajnc EI, Wester PW, Loeber JG, van Leeuwen FX, Vos JG, Vaessen HA (1984). Toxicity of bis(tri-n-butyltin)oxide in the rat. I. Short-term effects on general parameters and on the endocrine and lymphoid systems. Toxicol Appl Pharmacol.

[36] Appel KE (2004). Organotin compounds: Toxicokinetic aspects. Drug Metab Rev.

[37] Sena GC, Freitas-Lima LC, Merlo E, Podratz PL, de Araújo JF, Brandão PA (2017). Environmental obesogen tributyltin chloride leads to abnormal hypothalamic-pituitary-gonadal axis function by disruption in kisspeptin/leptin signaling in female rats. Toxicol Appl Pharmacol.

[38] Marques VB, Faria RA, Dos Santos L (2018). Overview of the pathophysiological implications of organotins on the endocrine system. Front Endocrinol (Lausanne).

[39] Nowak K, Jabłońska E, Ratajczak-Wrona W (2019). Immunomodulatory effects of synthetic endocrine disrupting chemicals on the development and functions of human immune cells. Environ Int.

[40] Gore AC, Chappell VA, Fenton SE, Flaws JA, Nadal A, Prins GS (2015). Executive Summary to EDC-2: The Endocrine Society's Second Scientific Statement on Endocrine-Disrupting Chemicals. Endocr Rev.

[41] Bergman, Åke, Heindel Jerrold J, World Health Organization, United Nations Environment Programme, Inter-Organization Programme for the Sound Management of Chemicals (2013). State of the science of endocrine disrupting chemicals 2012: summary for decision-makers.

[42] Zoeller RT, Brown TR, Doan LL, Gore AC, Skakkebaek NE, Soto AM (2012). Endocrine-disrupting chemicals and public health protection: a statement of principles from The Endocrine Society. Endocrinology.

[43] Delfosse V, Maire AL, Balaguer P, Bourguet W (2015). A structural perspective on nuclear receptors as targets of environmental compounds. Acta Pharmacol Sin.

[44] La Merrill MA, Vandenberg LN, Smith MT, Goodson W, Browne P, Patisaul HB (2020). Consensus on the key characteristics of endocrine-disrupting chemicals as a basis for hazard identification. Nat Rev Endocrinol.

[45] Bronowicka-Kłys DE, Lianeri M, Jagodziński PP (2016). The role and impact of estrogens and xenoestrogen on the development of cervical cancer. Biomed Pharmacother.

[46] Chamorro-Garcia R, Diaz-Castillo C, Shoucri BM, Käch H, Leavitt R, Shioda T (2017). Ancestral perinatal obesogen exposure results in a transgenerational thrifty phenotype in mice. Nat Commun.

[47] Chamorro-García R, Poupin N, Tremblay-Franco M, Canlet C, Egusquiza R, Gautier R (2021). Transgenerational metabolomic fingerprints in mice ancestrally exposed to the obesogen TBT. Environ Int.

[48] Penza M, Jeremic M, Marrazzo E, Maggi A, Ciana P, Rando G (2011). The environmental chemical tributyltin chloride (TBT) shows both estrogenic and adipogenic activities in mice which might depend on the exposure dose. Toxicol Appl Pharmacol.

[49] Grün F, Watanabe H, Zamanian Z, Maeda L, Arima K, Cubacha R (2006). Endocrine-Disrupting Organotin Compounds Are Potent Inducers of Adipogenesis in Vertebrates. Mol Endocrinol.

[50] Sharan S, Nikhil K, Roy P (2013). Effects of low dose treatment of tributyltin on the regulation of estrogen receptor functions in MCF-7 cells. Toxicol Appl Pharmacol.

[51] Chen N, Luo L, Zhang C, Liu J, Wang W, Li Y (2020). Anti-Müllerian hormone participates in ovarian granulosa cell damage due to cadmium exposure by negatively regulating stem cell factor. Reprod Toxicol.

[52] de Araújo JFP, Podratz PL, Sena GC, Merlo E, Freitas-Lima LC, Ayub JGM (2018). The obesogen tributyltin induces abnormal ovarian adipogenesis in adult female rats. Toxicol Lett.

[53] Atanasov AG, Nashev LG, Tam S, Baker ME, Odermatt A (2005). Organotins disrupt the 11beta-hydroxysteroid dehydrogenase type 2-dependent local inactivation of glucocorticoids. Environ Health Perspect.

[54] Shoucri BM, Hung VT, Chamorro-García R, Shioda T, Blumberg B (2018). Retinoid X receptor activation during adipogenesis of female mesenchymal stem cells programs a dysfunctional adipocyte. Endocrinology.

[55] Shoucri BM, Martinez ES, Abreo TJ, Hung VT, Moosova Z, Shioda T (2017). Retinoid x receptor activation alters the chromatin landscape to commit mesenchymal stem cells to the adipose lineage. Endocrinology.

[56] Ceotto Freitas-Lima L, Merlo E, Campos Zicker M, Navia-Pelaez JM, de Oliveira M, Dos Santos Aggum Capettini L (2018). Tributyltin impacts in metabolic syndrome development through disruption of angiotensin II receptor signaling pathways in white adipose tissue from adult female rats. Toxicol Lett.

[57] Chen M, Guo J, Ruan J, Yang Z, He C, Zuo Z (2020). Neonatal exposure to environment-relevant levels of tributyltin leads to uterine dysplasia in rats. Sci Total Environ.

[58] Woods L, Perez-Garcia V, Hemberger M (2018). Regulation of Placental Development and Its Impact on Fetal Growth – New Insights from Mouse Models. Front Endocrinol (Lausanne).

[59] Warner GR, Meling DD, De La Torre KM, Wang K, Flaws JA (2021). Environmentally relevant mixtures of phthalates and phthalate metabolites differentially alter the cell cycle and apoptosis in mouse neonatal ovaries. Biol Reprod.

[60] Grandjean P. (2008). Late insights into early origins of disease. Basic Clin Pharmacol Toxicol.

[61] Barker DJ, Clark PM (1997). Fetal undernutrition and disease in later life. Rev Reprod.

[62] Barker DJ (2004). The Developmental Origins of Adult Disease. J Am Coll Nutr.

[63] Schug TT, Janesick A, Blumberg B, Heindel JJ (2011). Endocrine disrupting chemicals and disease susceptibility. J Steroid Biochem Mol Biol.

[64] Woodruff TJ, Zota AR, Schwartz JM (2011). Environmental chemicals in pregnant women in the united states: NHANES 2003–2004. Environ Health Perspect.

[65] Johns LE, Ferguson KK, Cantonwine DE, McElrath TF, Mukherjee B, Meeker JD, Urinary BPA (2017). and phthalate metabolite concentrations and plasma vitamin D levels in pregnant women: A repeated measures analysis. Environ Health Perspect.

[66] Argyraki M, Damdimopoulou P, Chatzimeletiou K, Grimbizis GF, Tarlatzis BC, Syrrou M (2019). In-utero stress and mode of conception: Impact on regulation of imprinted genes, fetal development and future health. Hum Reprod Update.

[67] Liu H, Jiang W, Ye Y, Yang B, Shen X, Lu S (2021). Maternal exposure to tributyltin during early gestation increases adverse pregnancy outcomes by impairing placental development. Environ Toxicol.

[68] Nakanishi T, Hiromori Y, Yokoyama H, Koyanagi M, Itoh N, Nishikawa J (2006). Organotin compounds enhance 17beta-hydroxysteroid dehydrogenase type I activity in human choriocarcinoma JAr cells: Potential promotion of 17beta-estradiol biosynthesis in human placenta. Biochem Pharmacol.

[69] Adeeko A, Li D, Forsyth DS, Casey V, Cooke GM, Barthelemy J (2003). Effects of in utero tributyltin chloride exposure in the rat on pregnancy outcome. Toxicol Sci.

[70] Furukawa S, Tsuji N, Kobayashi Y, Yamagishi Y, Hayashi S, Abe M (2017). Effect of dibutyltin on placental and fetal toxicity in rat. J Toxicol Sci.

[71] Omura M, Ogata R, Kubo K, Shimasaki Y, Aou S, Oshima Y (2001). Two-generation reproductive toxicity study of tributyltin chloride in male rats. Toxicol Sci.

[72] Ogata R, Omura M, Shimasaki Y, Kubo K, Oshima Y, Aou S (2001). Two-generation reproductive toxicity study of tributyltin chloride in female rats. J Toxicol Environ Health A.

[73] Si J, Li J, Zhang F, Li G, Xin Q, Dai B (2012). Effects of perinatal exposure to low doses of tributyltin chloride on pregnancy outcome and postnatal development in mouse offspring. Environ Toxicol.

[74] Makita Y (2008). Effects of perinatal combined exposure to 1,1-dichloro-2,2-bis(p-chlorophenyl)ethylene (p,p′-DDE) and tributyltin (TBT) on rat female reproductive system. Environ Toxicol Pharmacol.

[75] Makita Y, Omura M (2006). Effects of perinatal combined exposure to 1,1-dichloro-2,2 bis (p-chlorophenyl) ethylene and tributyltin on male reproductive system. Basic Clin Pharmacol Toxicol.

[76] Si J, Li P, Xin Q, Li X, An L, Li J (2015). Perinatal exposure to low doses of tributyltin chloride reduces sperm count and quality in mice. Environ Toxicol.

[77] Shioda K, Odajima J, Blumberg B, Shioda T (2022). Transgenerational Transcriptomic and DNA Methylome Profiling of Mouse Fetal Testicular Germline and Somatic Cells after Exposure of Pregnant Mothers to Tributyltin, a Potent Obesogen. Metabolites.

[78] Kishta O, Adeeko A, Li D, Luu T, Brawer JR, Morales C (2007). In utero exposure to tributyltin chloride differentially alters male and female fetal gonad morphology and gene expression profiles in the Sprague-Dawley rat. Reprod Toxicol.

[79] Kariyazono Y, Taura J, Hattori Y, Ishii Y, Narimatsu S, Fujimura M (2015). Effect of in utero exposure to endocrine disruptors on fetal steroidogenesis governed by the pituitary-gonad axis: A study in rats using different ways of administration. J Toxicol Sci.

[80] Zanol JF, Niño OMS, da Costa CS, Freitas-Lima LC, Miranda-Alves L, Graceli JB (2021). Tributyltin and high-refined carbohydrate diet lead to metabolic and reproductive abnormalities, exacerbating premature ovary failure features in the female rats. Reprod Toxicol.

[81] Jannini EA, Ulisse S, D’Armiento M (1995). Thyroid Hormone and Male Gonadal Function. Endocr Rev.

[82] Buzzard JJ, Morrison JR, O’Bryan MK, Song Q, Wreford NG (2000). Developmental Expression of Thyroid Hormone Receptors in the Rat Testis. Biol Reprod.

[83] Blundell C, Yi YS, Ma L, Tess ER, Farrell MJ, Georgescu A (2018). Placental Drug Transport-on-a-Chip: A Microengineered In Vitro Model of Transporter-Mediated Drug Efflux in the Human Placental Barrier. Adv Healthc Mater.

[84] Fry RC, Bangma J, Szilagyi J, Rager JE (2019). Developing novel in vitro methods for the risk assessment of developmental and placental toxicants in the environment. Toxicol Appl Pharmacol.

[85] Gingrich J, Ticiani E, Veiga-Lopez A (2020). Placenta Disrupted: Endocrine Disrupting Chemicals and Pregnancy. Trends Endocrinol Metab.

[86] Grigsby PL (2016). Animal Models to Study Placental Development and Function throughout Normal and Dysfunctional Human Pregnancy. Semin Reprod Med.

[87] Gonçalves BM, Graceli JB, da Rocha PB, Tilli HP, Vieira EM, de Sibio MT (2022). Placental model as an important tool to study maternal-fetal interface. Reprod Toxicol.

